# Is metabolic-healthy obesity associated with risk of dementia? An age-stratified analysis of the Whitehall II cohort study

**DOI:** 10.1186/s12916-023-03155-4

**Published:** 2023-11-14

**Authors:** Marcos D. Machado-Fragua, Séverine Sabia, Aurore Fayosse, Céline Ben Hassen, Frank van der Heide, Mika Kivimaki, Archana Singh-Manoux

**Affiliations:** 1 Université Paris Cité, Inserm U1153, Epidemiology of Ageing and Neurodegenerative Diseases, Paris, France; 2 Faculty of Brain Sciences, University College London, London, UK

**Keywords:** BMI, Cardiometabolic risk factors, Obesity, Phenotypes, Metabolic status, Dementia

## Abstract

**Background:**

Metabolically healthy obesity is hypothesized to be a benign condition but whether this is the case for dementia remains debated. We examined the role of age at assessment of metabolic-obesity phenotypes in associations with incident dementia.

**Methods:**

Obesity (body mass index ≥ 30 kg/m^2^) and poor metabolic health (≥ 2 of elevated serum triglycerides, low HDL-C, elevated blood pressure, and elevated serum fasting glucose) were used to define four metabolic-obesity phenotypes (metabolically healthy (MHNO) and unhealthy non-obesity (MUNO), metabolically healthy (MHO) and unhealthy obesity (MUO)) at < 60, 60 to < 70, and ≥ 70 years using 6 waves of data from the Whitehall II study and their associations with incident dementia was examined using Cox regression.

**Results:**

Analyses with exposures measured < 60, 60 to < 70, and ≥ 70 years involved 410 (5.8%), 379 (5.6%), and 262 (7.4%) incident dementia cases over a median follow-up of 20.8, 10.3, and 4.2 years respectively. In analyses of individual components, obesity before 60 years (HR 1.41, 95% CI: [1.08, 1.85]) but not at older ages was associated with dementia; unhealthy metabolic status when present < 60 years (HR 1.33, 95% CI: [1.08, 1.62]) and 60 to < 70 years (HR 1.32, 95% CI: [1.07, 1.62]) was associated with dementia. Compared to the metabolically healthy non-obesity group, the risk of dementia was higher in those with metabolically healthy obesity before 60 years (1.69; 95% CI: [1.16, 2.45]); this was not the case when metabolic-obesity phenotype was present at 60 to < 70 years or ≥ 70 years. Analyses at older ages were on smaller numbers due to death and drop-out but inverse probability weighting to account for missing data yielded similar results.

**Conclusions:**

Individuals with metabolically healthy obesity before age 60 had a higher risk of incident dementia over a 27-year follow-up; the excess risk dissipates when metabolic health and obesity are measured after 70 years.

**Supplementary Information:**

The online version contains supplementary material available at 10.1186/s12916-023-03155-4.

## Background

Obesity, defined as body mass index (BMI) ≥ 30 kg/m^2^, is associated with poor health outcomes [1] but there is considerable heterogeneity in health outcomes in individuals with obesity. The concept of metabolically healthy obesity (MHO) [2] was elaborated to explain this heterogeneity. The hypothesis underlying this concept suggests that health outcomes in those with MHO are better than that among individuals with both obesity and poor metabolic health, i.e. metabolically unhealthy obesity (MUO). The most widely used metabolic-obesity phenotypes are based on obesity measured with BMI and the metabolic syndrome (MetS) [3, 4], the latter being a cluster of cardiometabolic components consisting of elevated waist circumference, elevated blood pressure, low high-density lipoprotein cholesterol (HDL-C), elevated triglycerides, and elevated fasting glucose.

Recent findings from longitudinal studies do not support the healthy obesity hypothesis as the risk of diabetes, respiratory diseases, and mortality [5] in individuals with MHO and MUO was found to be similar, and higher than in normal-weight individuals. Whether the same is true for old-age outcomes remains unclear as some studies reported no increased risk of all-cause dementia [6] and Alzheimer’s disease [6–8] in MHO persons compared to individuals without obesity. Dementia is a complex disorder with a long preclinical period involving multiple changes, such as weight loss in the years preceding diagnosis [9]. Pathophysiological processes in dementia may start 15 to 20 years before dementia diagnosis [10, 11], highlighting the importance of a long follow-up to separate the measurement of risk factors and onset of dementia. Accordingly, obesity and metabolic risk factors in mid-life have been found to be associated with higher dementia risk [12, 13] whereas obesity at older ages is not associated with a higher risk of dementia [14]. These findings highlight the importance of age at measurement of metabolic-obesity phenotypes for their association with dementia. Previous studies [6–8] that have examined this association used a wide age range at assessment of metabolic-obesity phenotypes, not allowing conclusions to be drawn on the effect of age (or length of the follow-up period) for associations with dementia.

Using measurements of weight, height, and metabolic factors at < 60, 60 to < 70, and ≥ 70 years, the objective of the present study was to examine the role of age at assessment of metabolic-obesity phenotypes in associations with incidence of dementia. In complementary analyses, we also examined associations with cognitive decline.

## Methods

### Study population

Data were drawn from the ongoing Whitehall II study where all men and women aged 35–55 working in the London offices of twenty civil-service departments were invited to the study with no inclusion/exclusion criteria; 10,308 were recruited in 19,585–1988, and the response rate was 73% [15]. The baseline consisted of a clinical examination and a standard self-administered questionnaire. The clinical examination is undertaken by research nurses who follow a protocol elaborated by the research team, with examination undertaken in central London premises hired for this purpose or at participants’ homes for those unable to travel to London. Each wave takes around 2 years to complete; follow-up clinical examinations have taken place approximately every 4 to 5 years since baseline (1991, 1997, 2002, 2007, 2012, 2015, and 2020) using the same protocol. Linkage to electronic health records of the UK National Health Service (NHS) was used to obtain records of health outcomes until March 31, 2019. Participants’ written informed consent and research ethics approval were renewed at each contact; the latest was from the Joint UCL/UCLH Committee on the Ethics of Human Research (reference number 85/0938).

### Metabolic-obesity phenotypes

Components of the metabolic-obesity phenotypes were measured six times (1991, 1997, 2002, 2007, 2012, and 2015) for each participant and data were extracted from measurements taken at < 60 (range: 40 to 59.9 years), 60 to < 70 (range: 60 to 69.9 years), and ≥ 70 (range: 70 to 84 years) using multiple waves of the study (Additional file 1: Fig. S1). When data were available at several time points within an age category, the measure closest to age 55, 65, and 75 years was chosen for the three groups, respectively.

Weight was measured to the nearest 0.1 kg on digital Soehnle electronic scales with participants in light clothing. Height was measured to the nearest 1 mm using a stadiometer with participants standing erect in bare feet with the head in the Frankfurt plane. BMI was calculated as weight (kg) divided by height (m) squared in kg/m^2^. Participants were classified based on their BMI as non-obesity (BMI < 30 kg/m^2^) or obesity (BMI ≥ 30 kg/m^2^) [16]. Underweight participants (BMI < 18.5 kg/m^2^) were removed from the analyses. Metabolic health was measured using components of the metabolic syndrome [17]. As in previous studies [6–8], the waist circumference criterion was excluded due to collinearity with BMI (variance inflation factor > 100 for both BMI and waist circumference in all study waves, suggesting high collinearity).

Poor metabolic status was defined as a prevalence of ≥ 2 of the following criteria: (a) elevated serum triglycerides (≥ 150 mg/dL [1.7 mmol/L], or use of lipid-modifying drugs); (b) low HDL-C (in men: < 40 mg/dL [1.0 mmol/L] and in women: < 50 mg/dL [1.3 mmol/L], or use of lipid-modifying drugs); (c) elevated blood pressure (systolic blood pressure ≥ 130 mmHg and/or diastolic blood pressure ≥ 85 mmHg, or use of antihypertensive drugs), blood pressure was the mean of two measurements using a sphygmomanometer with the participant in a sitting position after 5 min of rest; and (d) elevated serum fasting glucose (≥ 100 mg/dL [5.6 mmol/L]) or use of glucose-lowering drugs.

Blood samples were handled according to standard protocols, with participants in a fasting state (≥ 8 h fasting or ≥ 5 h for afternoon visits). Venipuncture of the antecubital vein in the left arm used to draw blood, collected in plain and fluoride Sarstedt (Neumbrecht, Germany) monovettes. Plasma or serum was immediately moved into microtubes and stored at – 70 °C. HDL-C was measured by precipitating non-HDL-cholesterol with dextran sulfate-magnesium chloride using a centrifuge and measuring cholesterol in the supernatant fluid. Serum triglycerides were determined by the enzymatic colorimetric method (glycerol-3-phosphate oxidase/phenol and aminophenazone). Serum glucose was measured using the glucose oxidase method (YSI MODEL 2300 STAT PLUS Analyzer, YSI Corporation, Yellow Springs, OH, USA) [18]. The assays were performed by research-accredited laboratories in the London area; technical error was estimated by assaying blinded duplicate samples for 5% of subjects and coefficients of variation were 2.0 to 6.6%.

Metabolic-obesity phenotypes were defined based on obesity (yes/no) and poor metabolic status (yes/no) and included: metabolically healthy non-obesity (MHNO), metabolically unhealthy non-obesity (MUNO), metabolically healthy obesity (MHO) and metabolically unhealthy obesity (MUO).

### Dementia

Dementia was ascertained by linkage to three electronic health records databases (the national Hospital Episode Statistics (HES), the Mental Health Services Data Set (MHSDS), and the National Statistics Mortality Register) until March 31, 2019. Dementia cases were identified based on ICD-10 codes F00-F03, F05.1, G30, and G31. The NHS provides most of the health care in the UK, including in- and outpatient care. Ascertainment of all-cause dementia using the HES data has a sensitivity and specificity of 78.0% and 92.0% [19]. The sensitivity in our study is likely higher as we also used data from the MHSDS and the mortality register. The date of dementia was defined as the earliest date at which dementia had been diagnosed via any register.

### Cognitive test battery

The cognitive function test battery from measurements in 1997, 2002, 2007, 2012, and 2015 was used in the analyses. The battery included tests of (a) memory, assessed using a 20-word free recall test where a list of one or two-syllable words was presented to participants who then had to write as many words as they could recall within 2 min; (b) reasoning, assessed in 10 min via the Alice Heim 4-I test [20], which is composed of a series of 65 verbal and mathematical items of increasing difficulty, and inductive reasoning tests by measuring the ability to identify patterns and infer principles and rules; and (c) phonemic and semantic fluency, where participants were asked to recall in writing as many words beginning with “s” (phonemic fluency) and as many animal names (semantic fluency) as they could in one minute for each test.

Individual test scores at each wave were standardized to a *z*-score (mean 0, standard deviation 1) using the mean and standard deviation of the baseline 1997 measure. In addition, a global cognitive score was created by averaging all four standardized tests and then re-standardizing the resulting score, leading to a score of mean 0 and standard deviation of 1. The global z-score is useful as it minimizes measurement error inherent in each individual test [21].

### Covariates

Sociodemographic factors included age, sex, ethnicity (white and non-white), education (high, intermediate, or low), and marital status (married, widowed, and single). Health-related behaviors included were smoking (never, former, and current smoker), alcohol consumption (no consumption, 1–14 units per week, and > 14 units per week), consumption of fruits and vegetables (less than daily, once a day, and twice or more a day) and time spent in moderate and vigorous physical activity (hours per week). Cardiovascular disease (CVD) was defined as a history of stroke (assessed with the MONICA-Ausburg stroke questionnaire; and from ICD-10 codes I60–64), coronary heart disease (CHD; assessed from 12-lead resting electrocardiogram recording; and from ICD-10 codes I20–25), and/or heart failure (assessed from ICD-10 code I50). Data on covariates were extracted in a similar manner as the metabolic-obesity phenotypes at < 60, 60 to < 70, and ≥ 70 years, and were concurrent to the measure of these phenotypes for each analysis.

### Statistical analysis

We tested whether the associations of metabolic-obesity phenotypes with cognitive decline and dementia varied by sex and found no evidence of differences (all p for interaction > 0.05), leading us to combine men and women in the analyses.

#### Association of metabolic-obesity phenotypes with incident dementia

We first examined the association of obesity, poor metabolic health, and components of metabolic status (at < 60, 60 to < 70, and ≥ 70 years) with dementia using Cox proportional hazards regression with age as the timescale. The proportional hazards assumption was examined by plotting Schoenfeld residuals and found not to be violated in the analyses. The beginning of the follow-up for incident dementia was age at assessment of the exposure (at < 60, 60 to < 70, and ≥ 70 years), prevalent dementia cases at the start of the follow-up were excluded. Participants were censored at the date of record of dementia, death, or end of follow-up (March 31, 2019), whichever occurred first. Cause-specific hazard models were used to account for competing risk of death. All analyses were first adjusted for sociodemographic factors and birth-cohort effects using 5-year bands of birth-year (model 1), and then for health-related behaviors (model 2) and mutually adjusted (model 3). We then examined the association of metabolic-obesity phenotypes (4 groups with MHNO phenotype as the reference) with incidence of dementia using the approach described above, without model 3.

We performed additional analyses to examine the robustness of our findings. First, we used inverse probability weighting to repeat the main analyses to take missing data into account. Attrition over the study period led to analyses on a smaller number of participants in the analysis on exposure measured at older ages. Inverse probability weighting allowed us to check if the results are affected by missing data [22]. This involved first calculating the probability of being included in the analytical sample using logistic regression, in a model that included demographic (age, sex, and ethnicity), socioeconomic (educational level, occupation, and marital status), behavioral factors (physical activity, smoking status, alcohol consumption, fruit and vegetables consumption, 28-item General Health Questionnaire, SF-36 Physical, and Mental Health Summary Scales), as well as BMI and metabolic components at the 1991 wave, chronic diseases (CHD, stroke, diabetes, chronic obstructive pulmonary disease, cancer, and dementia) during the follow-up, and stepwise-selected interactions between covariates. The inverse of these probabilities was used as weights in the Cox regression. Weights were calculated separately for each age-stratified analysis at ages < 60, 60 to < 70, and ≥ 70 years. Second, as ethnic groups may have different thresholds of obesity [16], we repeated the analyses on only “white” participants; the non-white group was too small to allow further analyses in this group. Third, to examine the role of prevalent CVD in our analyses, we excluded participants with CVD at baseline in the analyses. Fourth, as even one metabolic abnormality may affect dementia risk [23], we repeated the main analyses using an alternative definition with unhealthy metabolic status defined as the prevalence of ≥ 1 instead of ≥ 2 metabolic components.

#### Association of metabolic-obesity phenotypes with cognitive decline over 18 years

In complementary analyses, we examined the association of metabolic-obesity phenotype components (separate models) and phenotypes (4 groups) at the 1997 wave of data collection with cognitive decline between 1997 and 2015 using linear mixed models [24], with time of follow-up as the timescale. These models consider the fact that repeated measures on the same individual are correlated, and use all available data during the follow-up period. Individual differences in cognitive performance in 1997 and the rate of cognitive decline were estimated by fitting both the intercept and slope as random effects. The analyses included terms for metabolic-obesity phenotype, time of follow-up, time^2^, and the interaction of metabolic-obesity phenotype with time terms, and were adjusted for age and sociodemographic factors (Model 1), and then for health-related behaviors (Model 2) at the baseline in these analyses (1997 wave). All models included interactions of covariates with time, and interactions of covariates with time^2^ when *p* < 0.05.

#### Sensitivity analyses: association of trajectories of metabolic-obesity phenotypes between 1991 and 2002 with incidence of dementia (2002 to 2019) and 12-year cognitive decline (2002 to 2015)

While the focus of the main analyses was on the role of age at measurement of metabolic-obesity phenotypes we examined the association between trajectories of these phenotypes and both incident dementia and cognitive decline in sensitivity analyses. To allow sufficient follow-up for dementia we used data on metabolic-obesity phenotypes from measures in 1991, 1997, and 2002 to construct trajectories using group-based trajectory modeling [25]. The STATA Traj package’s censored normal model was used for these analyses. To determine the optimal number of trajectories, analyses were repeated to obtain 4 to 6 trajectories. Linear and quadratic functional forms were used to choose the best-fitting models, and the optimal trajectory shape and number of groups were determined based on the following criteria: (1) the number of participants within each trajectory group (≥ 5% of the total sample size); (2) the average posterior probability of each trajectory group (≥ 0.70); and (3) the lowest BIC/AIC value [26, 27]. The trajectories identified by these analyses were used as the exposure in Cox regression (for incident dementia) and linear mixed models (for cognitive decline between 2002 and 2015); covariates in these analyses were drawn from the 2002 wave.

Analyses were undertaken using STATA version 16.1 (StataCorp). A two-sided *p* value < 0.05 was considered statistically significant.

## Results

### Association of metabolic-obesity phenotypes with incident dementia

Of the 10,308 participants recruited to the study in 1985, 159 (1.5%) died before 1991, and 1708 (16.6%) had dropped out of the study when they were 40 to 59.9 years. We also excluded 1274 (12.4%) participants with missing data on metabolic-obesity phenotypes, three participants with missing data on covariates, and 55 (0.5%) participants with BMI < 18.5 kg/m^2^, leading to 7109 (69.0%) participants free of dementia in the analyses of exposures measured before 60 years (mean age at clinical examination 55.1 years, standard deviation [SD] 3.0 years; Additional file 1: Fig. S1).

The analyses on metabolic-obesity phenotypes measured < 60 years had a mean follow-up of 19.7 (SD = 6.1) years and 410 (5.8%) incident dementia cases were recorded. The mean age at dementia diagnosis was 76.5 (SD = 5.8) years. Additional file 1: Fig. S1 describes the sample selection in the analysis for metabolic-obesity phenotypes measured at ages < 60 years, 60 to < 70 years, and ≥ 70 years. Participants’ characteristics at ages < 60, 60 to < 70, and ≥ 70 years, as a function of dementia status at the end of follow-up are shown in Table 1. Compared with dementia-free participants, those who developed dementia during follow-up were more likely to be women, of non-white ethnicity, had a lower educational level, and consumed less fruit and vegetables.

**Table 1:** Sample characteristics at < 60, 60 to < 70, and ≥ 70 years overall and according to dementia status at the end of follow-up (31 March 2019)

	**At age < 60 years**				**At ages 60 years to < 70 years**				**At age ≥ 70 years**			
	**Total population**	**Dementia**		***p***	**Total population**	**Dementia**		***p***	**Total population**	**Dementia**		***p***
		**No**	**Yes**			**No**	**Yes**			**No**	**Yes**	
Age, M(SD)	55.0 (3.0)	55.0 (3.0)	56.3 (2.6)	< 0.01	65.0 (1.5)	65.0 (1.5)	64.9 (1.7)	0.33	73.9 (1.9)	73.9 (1.9)	74.2 (1.9)	0.01
Sex, women	2160 (30.4)	2007 (30.0)	153 (37.3)	< 0.01	1849 (28.8)	1717 (28.5)	132 (34.8)	0.01	1036 (29.1)	945 (28.6)	91 (34.7)	0.04
Education, low	3179 (44.7)	2950 (44.0)	229 (55.9)	< 0.01	2791 (43.5)	2579 (42.8)	212 (55.9)	< 0.01	1747 (49.0)	1594 (48.3)	153 (58.4)	< 0.01
Ethnicity, non-white	711 (10.0)	647 (9.7)	64 (15.6)	< 0.01	543 (8.5)	488 (8.1)	55 (14.5)	< 0.01	346 (9.7)	307 (9.3)	39 (14.9)	< 0.01
Marital status, married	5426 (76.3)	5125 (76.5)	301 (73.4)	0.44	4889 (76.3)	4616 (76.5)	273 (72.0)	0.20	2564 (71.9)	2400 (72.7)	164 (62.6)	0.01
Smoking, current smokers	842 (11.8)	789 (11.8)	53 (12.9)	0.46	414 (6.5)	383 (6.4)	31 (8.2)	0.05	107 (3.0)	102 (3.1)	5 (1.9)	0.52
Alcohol, moderate drinkers	3795 (53.4)	3608 (53.9)	187 (45.6)	< 0.01	3450 (53.8)	3262 (54.1)	188 (49.6)	< 0.01	1964 (55.1)	1836 (55.6)	128 (48.9)	< 0.01
Fruits and vegetables, ≥ 2/day	2171 (30.5)	2091 (31.2)	80 (19.5)	< 0.01	3018 (47.1)	2893 (48.0)	125 (33.0)	< 0.01	1786 (50.1)	1700 (51.5)	86 (32.8)	< 0.01
PA, h/week, M(SD)	3.3 (3.6)	3.3 (3.6)	3.0 (3.9)	< 0.01	4.0 (3.6)	4.0 (3.6)	3.4 (3.3)	< 0.01	3.5 (3.3)	3.6 (3.3)	3.2 (3.3)	0.02
Prevalence of CVD	383 (5.4)	357 (5.3)	26 (6.3)	0.38	774 (12.1)	721 (12.0)	53 (14.0)	0.24	799 (22.4)	725 (22.0)	74 (28.2)	0.02
Body mass index, M(SD)	26.3 (4.0)	26.3 (4.0)	26.3 (4.1)	0.31	26.8 (4.3)	26.8 (4.3)	26.6 (4.2)	0.30	26.9 (4.3)	26.9 (4.3)	26.7 (4.6)	0.22
**Metabolic components**												
Elevated triglycerides	2184 (30.7)	2057 (31.0)	127 (31.0)	0.91	2559 (39.9)	2421 (40.1)	138 (36.4)	0.15	1914 (53.7)	1771 (53.6)	143 (54.6)	0.77
Low HDL-C	1069 (15.0)	986 (14.7)	83 (20.2)	< 0.01	1904 (29.7)	1802 (29.9)	102 (26.9)	0.22	1748 (49.1)	1619 (49.0)	129 (49.2)	0.95
Elevated blood pressure	3187 (44.8)	2970 (44.3)	217 (52.9)	< 0.01	3756 (58.6)	3534 (58.6)	222 (58.6)	0.99	2581 (72.4)	2386 (72.3)	195 (74.4)	0.45
Elevated fasting glucose	1636 (23.0)	1538 (23.0)	98 (24.0)	0.64	1682 (26.2)	1568 (26.0)	114 (30.1)	0.08	976 (27.4)	884 (26.8)	92 (35.1)	< 0.01

Date are *n* (%), unless otherwise specified

*M* mean, *S*D standard deviation, *PA* moderate-vigorous physical activity, *CVD* cardiovascular disease (stroke, coronary heart disease, and heart failure), *HDL-C* high-density lipoprotein-cholesterol

Obesity and unhealthy metabolic status measured before 60 years, and considered separately, were associated with dementia; the mutually adjusted hazard ratios (HR) being 1.31 (95% CI: [1.00, 1.73]) and 1.27 (95% CI: [1.02, 1.57]), respectively (Additional file 1: Table S1). Unhealthy metabolic status (HR 1.33, 95%: CI [1.07, 1.64]) but not obesity measured between 60 and 70 years was also associated with a higher risk of dementia. No associations were found for measures of these exposures after age 70, when the median follow-up was 4.2 years. Among the individual components only fasting glucose after age 70 was associated with dementia in the mutually adjusted model (HR 1.38, 95% CI: [1.06, 1.81]).

The associations between metabolic-obesity phenotypes measured at ages < 60, 60 to < 70, and ≥ 70 years and incidence of dementia over a median follow-up of 20.8, 10.3, and 4.2 years, respectively are shown in Table 2. Compared to the MHNO group, the risk of dementia was higher in the MUNO (HR 1.38, 95% CI: [1.10, 1.73]), MHO (HR 1.69, 95% CI: [1.16, 2.45]), and MUO (HR 1.46, 95% CI: [1.02, 2.08]) groups when these phenotypes were measured before 60 years in analyses adjusted for all covariates. Pairwise comparisons, retaining all 4 groups in the analyses and changing the reference category showed the HR in the MHO and MUO group, defined at < 60 years, not to be different from each other (*p* = 0.50). Analysis using a measure of phenotypes at 60 to < 70 years showed higher HR in the MUNO (HR 1.28, 95% CI: [1.01, 1.61]) and MUO (HR 1.47, 95% CI: [1.05, 2.04]) groups but not the MHO group (HR 1.02, 95% CI: [0.65, 1.58]). No associations were found when these phenotypes were measured at age ≥ 70 years.

**Table 2:** Association of metabolic-obesity phenotypes at < 60, 60 to < 70, and ≥ 70 years with incidence of dementia

	**Metabolic-obesity phenotypes**			
	**MHNO**	**MUNO**	**MHO**	**MUO**
**At age < 60 years**^**a**^**, median (IQR) follow-up 20.8 (15.5, 26.4) years**				
*N* dementia cases/total	217/4301	123/1715	33/503	37/590
Rate/1000 person-years	2.51	3.64	3.62	3.46
Models, HR (95% CI)				
Model 1^d^	Ref	1.43 (1.14, 1.79)	1.71 (1.18, 2.49)	1.54 (1.09, 2.20)
Model 2^e^	Ref	1.38 (1.10, 1.73)	1.69 (1.16, 2.45)	1.46 (1.02, 2.08)
**At ages 60 years to < 70 years**^**b**^**, median (IQR) follow-up 10.3 (6.3, 15.4) years**				
*N* dementia cases/total	183/3117	127/2067	23/433	46/795
Rate/1000 person-years	5.20	5.92	5.02	6.11
Models, HR (95% CI)				
Model 1^d^	Ref	1.28 (1.02, 1.62)	1.06 (0.68, 1.64)	1.54 (1.11, 2.14)
Model 2^e^	Ref	1.28 (1.01, 1.61)	1.02 (0.65, 1.58)	1.47 (1.05, 2.04)
**At age ≥ 70 years**^**c**^**, median (IQR) follow-up 4.2 (3.1, 7.1) years**				
*N* dementia cases/total	83/1244	121/1589	14/191	44/540
Rate/1000 person-years	11.33	13.24	13.18	14.63
Models, HR (95% CI)				
Model 1^d^	Ref	1.18 (0.89, 1.56)	1.09 (0.61, 1.94)	1.28 (0.88, 1.85)
Model 2^e^	Ref	1.16 (0.87, 1.53)	0.99 (0.56, 1.78)	1.20 (0.82, 1.74)

*MHNO* metabolically healthy non-obesity, *MUNO* metabolically unhealthy non-obesity, *MHO* metabolically healthy obesity, *MUO* metabolically unhealthy obesity, *IQR* interquartile range, *HR* hazard ratio, *CI* confidence interval, *NA* not applicable

^a^Mean (SD) age at assessment = 55.1 (2.9) years

^b^Mean (SD) age at assessment = 65.0 (1.5) years

^c^Mean (SD) age at assessment = 73.9 (1.9) years

^d^Model 1: analyses adjusted for age (timescale), sex, education, ethnicity, marital status, and birth cohort (5-year groups)

^e^Model 2: Model 1 plus adjustment for health-related behaviors (smoking, alcohol consumption, consumption of fruits and vegetables, and physical activity)

Analyses using inverse probability weighting to account for missing data (Additional file 1: Table S2), as well as the exclusion of non-white participants (Additional file 1: Table S3) and prevalent cases of CVD (Additional file 1: Table S4), yielded results similar to that in the main analyses. Further analyses with an alternative threshold (≥ 1 instead of ≥ 2 unhealthy metabolic components) show a similar pattern to that in the main analyses (Additional file 1: Table S5).

The change in MHO status across the 3 age groups is shown in Additional file 1: Table S6. In the 503 participants with MHO < 60 years, 30.6% remained MHO at the 60 to < 70 years measure and 31.4% transitioned to MUO status. In 433 participants categorized as MHO at 60 to < 70 years, 20.3% remained MHO and 23.6% transitioned to the MUO phenotype at the ≥ 70 years measure.

### Association of metabolic-obesity phenotypes with 18-year cognitive decline

Of the 8097 participants at the 1997 wave (the baseline in these analyses), data were missing for 406 (5.0%) participants on metabolic-obesity phenotypes, for 531 (6.6%) participants on cognitive tests, for one participant on covariates, resulting in 7156 (88.4%) participants in the analyses on cognitive decline (Additional file 1: Fig. S2), their characteristics at baseline are shown in Additional file 1: Table S7. Compared with the MHNO phenotype, the MHO and MUO groups experienced an accelerated cognitive decline in the global cognitive score (Table 3); the MHO group also experienced a faster decline in reasoning, semantic fluency, and phonemic fluency in the fully adjusted analyses (all *p* < 0.05).

**Table 3:** Cognitive decline over 18 years as a function of metabolic-obesity phenotypes in 1997

**Standardized cognitive test scores**	**Metabolic-obesity phenotypes in 1997**^a^			
	**MHNO (*N* = 4726)** **Reference** **Mean (95% CI)**	**MUNO (*N* = 1465)** **Difference (95% CI)**	**MHO (*N* = 481)** **Difference (95% CI)**	**MUO (*N* = 484)** **Difference (95% CI)**
**Global cognitive score**^**b**^				
Model 1^c^	− 0.71 (− 0.73, − 0.69)	− 0.01 (− 0.06, 0.03)	− 0.15 (− 0.22, − 0.07)	− 0.10 (− 0.18, − 0.02)
Model 2^d^	− 0.72 (− 0.74, − 0.69)	− 0.01 (− 0.06, 0.04)	− 0.14 (− 0.22, − 0.07)	− 0.10 (− 0.17, − 0.02)
**Individual cognitive tests**				
**Memory**				
Model 1^c^	− 0.73 (− 0.76, − 0.70)	0.04 (− 0.03, 0.10)	− 0.08 (− 0.19, 0.03)	− 0.12 (− 0.22, 0.01)
Model 2^d^	− 0.73 (− 0.76, − 0.70)	0.04 (− 0.03, 0.11)	− 0.07 (− 0.18, 0.04)	− 0.10 (− 0.21, 0.01)
**Reasoning**				
Model 1^c^	− 0.43 (− 0.45, − 0.41)	− 0.03 (− 0.07, 0.01)	− 0.09 (− 0.15, − 0.03)	− 0.05 (− 0.12, 0.01)
Model 2^d^	− 0.43 (− 0.45, − 0.42)	− 0.03 (− 0.07, 0.01)	− 0.09 (− 0.16, − 0.03)	− 0.06 (− 0.12, 0.01)
**Semantic fluency**				
Model 1^c^	− 0.46 (− 0.49, − 0.43)	− 0.02 (− 0.08, 0.03)	− 0.09 (− 0.18, − 0.01)	0.01 (− 0.08, 0.10)
Model 2^d^	− 0.46 (− 0.49, − 0.44)	− 0.02 (− 0.08, 0.03)	− 0.09 (− 0.18, − 0.004)	0.002 (− 0.09, 0.09)
**Phonemic fluency**				
Model 1^c^	− 0.51 (− 0.54, -0.48)	− 0.01 (− 0.07, 0.05)	− 0.17 (− 0.27, − 0.08)	− 0.16 (− 0.26, − 0.06)
Model 2^d^	− 0.52 (− 0.54, − 0.49)	− 0.01 (− 0.07, 0.05)	− 0.17 (− 0.26, − 0.07)	− 0.16 (− 0.26, − 0.06)

*MHNO* metabolically healthy non-obesity, *MUNO* metabolically unhealthy non-obesity, *MHO* metabolically healthy obesity, *MUO* metabolically unhealthy obesity, *BMI* body mass index

^a^Defined as prevalence of ≥ 2 of elevated blood pressure, elevated triglycerides, low HDL-C, and/or elevated fasting glucose

^b^The global cognitive score is composed of all 4 cognitive tests, re-standardized to a *z*-score

^c^Model 1: Analysis adjusted for time, time^2^, age, sex, education, ethnicity, marital status, and interactions with time and time^2^ when *p* < 0.05

^d^Model 2: Model 1 + health-related behaviors (smoking, alcohol consumption, fruits and vegetables consumption, physical activity), and interactions with time and time^2^ when *p* < 0.05

### Sensitivity analyses: association of trajectories of metabolic-obesity phenotypes between 1991 and 2002 with incidence of dementia (2002 to 2019) and 12-year cognitive decline (2002 to 2015).

Five trajectories of metabolic-obesity phenotypes were identified (Additional file 1: Tables S8 and S9): persistent MHNO (45% of participants were in this group), transition from MUNO to MHNO (5%), transition from MHNO to MUNO (9%), persistent/transition to unhealthy metabolic status and/or transition to obesity (32%), and transition to obesity or persistent obesity (9%). Compared with participants in the persistent MHNO group, those in the transition to obesity or persistent obesity group had a higher risk of incident dementia (HR 1.40, 95% CI: [1.01, 1.95]; Table 4) and faster 12-year cognitive decline in the global cognitive score and all individual cognitive domains, except semantic fluency (Table 5), in analyses adjusted for all covariates.

**Table 4:** Association of metabolic-obesity phenotypes trajectories between 1991 and 2002 with risk of dementia

Metabolic-obesity phenotypes trajectories	*N* dementia cases/total	Dementia rate per 1000 person-years	Hazard ratio (95% CI)	
			**Model 1**^**a**^	**Model 2**^**b**^
**Median follow-up 15.5 (IQR 15.0–15.9) years**				
Persistent MHNO	149/3048	3.30	Ref	Ref
Transition from MUNO to MHNO	18/321	3.93	0.94 (0.58, 1.54)	0.87 (0.52, 1.43)
Transition from MHNO to MUNO	29/603	3.28	0.86 (0.58, 1.29)	0.87 (0.58, 1.29)
Persistent/transition to unhealthy metabolic status and/or transition to obesity	169/2195	5.41	1.27 (1.02, 1.59)	1.22 (0.98, 1.53)
Transition to obesity and persistent obesity	50/622	5.77	1.52 (1.10, 2.11)	1.40 (1.01, 1.95)

*MHNO* metabolically healthy non-obesity, *MUNO* metabolically unhealthy non-obesity

^a^Model 1 indicates that analyses were adjusted for age (as timescale), sex, education, ethnicity, marital status, and birth cohort (5-year groups)

^b^Model 2 indicates model 1 adjustment plus adjustment for health-related behaviors (smoking, alcohol consumption, diet, and physical activity)

**Table 5:** Association of metabolic-obesity phenotypes trajectories between 1991 and 2002 with subsequent 12-year cognitive decline

Standardized cognitive test scores	Metabolic-obesity phenotypes trajectories				
	**Persistent MHNO** **Reference** **Mean (95% CI)**	**Transition from MUNO to MHNO** **Difference (95% CI)**	**Transition from MHNO to MUNO** **Difference (95% CI)**	**Persistent/transition to unhealthy metabolic status and/or transition to obesity** **Difference (95% CI)**	**Transition to obesity and persistent obesity** **Difference (95% CI)**
**Global cognitive score**^**a**^					
Model 1^b^	− 0.44 (− 0.46, − 0.41)	− 0.01 (− 0.09, 0.06)	0.02 (− 0.04, 0.08)	− 0.04 (− 0.08, 0.002)	− 0.12 (− 0.18, − 0.05)
Model 2^c^	− 0.44 (− 0.46, − 0.41)	− 0.01 (− 0.09, 0.07)	0.02 (− 0.04, 0.08)	− 0.04 (− 0.08, 0.004)	− 0.11 (− 0.18, − 0.05)
**Individual cognitive tests**					
**Memory**					
Model 1^b^	− 0.61 (− 0.65, − 0.57)	− 0.05 (− 0.17, 0.07)	− 0.001 (− 0.09, 0.09)	− 0.02 (− 0.09, 0.04)	− 0.14 (− 0.24, − 0.04)
Model 2^c^	− 0.61 (− 0.65, − 0.58)	− 0.05 (− 0.17, 0.07)	0.001 (− 0.09, 0.09)	− 0.02 (− 0.08, 0.04)	− 0.14 (− 0.24, − 0.04)
**Reasoning**					
Model 1^b^	− 0.21 (− 0.23, − 0.19)	0.01 (− 0.06, 0.07)	0.04 (− 0.01, 0.09)	− 0.03 (− 0.06, 0.001)	− 0.07 (− 0.12, − 0.01)
Model 2^c^	− 0.21 (− 0.23, − 0.19)	0.01 (− 0.06, 0.08)	0.04 (− 0.01, 0.09)	− 0.03 (− 0.06, 0.01)	− 0.06 (− 0.11, − 0.003)
**Semantic fluency**					
Model 1^b^	− 0.21 (− 0.24, − 0.18)	− 0.04 (− 0.14, 0.06)	0.06 (− 0.02, 0.14)	− 0.02 (− 0.07, 0.04)	0.01 (− 0.08, 0.09)
Model 2^c^	− 0.21 (− 0.25, − 0.18)	− 0.04 (− 0.14, 0.06)	0.06 (− 0.02, 0.14)	− 0.02 (− 0.07, 0.04)	0.01 (− 0.08, 0.09)
**Phonemic fluency**					
Model 1^b^	− 0.29 (− 0.32, − 0.25)	0.06 (− 0.04, 0.17)	− 0.02 (− 0.10, 0.07)	− 0.04 (− 0.09, 0.02)	− 0.13 (− 0.22, − 0.04)
Model 2^c^	− 0.29 (− 0.32, − 0.25)	0.06 (− 0.05, 0.17)	− 0.02 (− 0.10, 0.07)	− 0.04 (− 0.09, 0.02)	− 0.13 (− 0.22, − 0.04)

MHNO metabolically healthy non-obesity, MUNO metabolically unhealthy non-obesity

^a^The global cognitive score is composed of all 4 cognitive tests, re-standardized to a *z*-score

^b^Model 1: Analysis adjusted for time, time^2^, age, sex, education, ethnicity, marital status, birth cohort (5-year groups), and interactions with time and time^2^ when *p* < 0.05

^c^Model 2: Model 1 + health-related behaviors (smoking, alcohol consumption, fruits and vegetables consumption, physical activity), and interactions with time and time^2^ when *p* < 0.05

## Discussion

The primary finding from our prospective analyses based on objective measures of metabolic-obesity phenotypes and data spanning up to 27 years is that metabolically healthy obesity before age 60 is associated with a higher risk of incident dementia and accelerated 18-year cognitive decline. These results do not support the hypothesis that MHO is a benign condition, particularly when this condition is present before age 60. Obesity before but not after age 60 was associated with a higher risk of dementia, and individuals in the metabolically healthy and unhealthy obesity group had a similar, higher risk of dementia compared to individuals in the metabolically healthy non-obesity group. It is worth noting that across the 3 age strata, a considerable proportion of MHO individuals become MUO over the study follow-up. Furthermore, the “persistent obesity or transition to obesity” trajectory identified using repeat measures of metabolic-obesity phenotypes was also associated with a higher risk of incident dementia and faster cognitive decline compared with the “persistent metabolically-healthy non-obesity” trajectory.

There is a lack of consensus on the definition of metabolically healthy obesity phenotype, making comparisons between studies difficult. Our analyses are based on the most widely used definition, using BMI and MetS without the waist circumference criterion to avoid collinearity with BMI. A number of studies have examined the association of metabolic-obesity phenotypes with dementia using the same definition but none of them have considered the impact of age at assessment of metabolic-obesity phenotypes or the length of follow-up to consider the long preclinical phase of dementia. Pathophysiological changes in dementia begin 15 to 20 years before the onset of clinical symptoms, making the timing of assessment of risk factors important to minimize reverse causation bias. Three previous studies that examined the risk of all-cause dementia [6] or Alzheimer’s disease [6–8] are characterized by older age at baseline examination (mean age between 67.1 and 73.5) and a short follow-up (ranging from 5 to 10 years). Two of these are based on data from the Korean National Health Insurance System where the mean age of participants was 67.1 years at measurement of metabolic-obesity phenotypes. In the first of these studies based on 363,932 individuals were followed for 65 months, and rather surprisingly the risk of dementia and Alzheimer’s disease in MHO was lower than in the MHNO group [6]. This pattern was also observed in the second study using these data, based on 136,847 individuals where the authors show a reduction in risk of Alzheimer’s disease in individuals who remained MHO over time [7]. The third study based on 1199 participants from the Alzheimer’s Disease Neuroimaging Initiative (ADNI) cohort, mean age 73.5 years at baseline, also reported the lowest risk of Alzheimer’s disease in the MHO group [8].

The studies described above suggest that obesity accompanied by good metabolic health is the profile that carries the lowest risk for dementia. These findings are consistent with the so-called obesity paradox hypothesis, elaborated in studies on the association between obesity and dementia that show late-life obesity to have a protective association with dementia [14]. It is worth noting that studies with repeat BMI data across the adult lifecourse show obesity in midlife to be associated with a higher risk of dementia, with an attenuation or reversal of the association when obesity is measured at older ages [9, 28]. The preclinical period of dementia is characterized by a number of changes, including weight loss, and results from studies where obesity is measured at older ages may simply reflect reverse causation. This view is in line with recent guidelines highlighting mid- rather than late-life obesity to be a risk factor for dementia [12]. Our results analyses using a median follow-up of 20.8 years (phenotypes measured before age 60) show MHO to be associated with a higher risk of dementia. When measured after the age of 70 (median follow-up 4.2 years), metabolic-obesity phenotypes had no association with dementia; these results are unlikely to be due to selection bias as inverse probability weighting to consider missing data yielded similar results to those in the main analysis. Although it was not possible to compare estimates across age strata formally as these are not sub-groups and most individuals are included in all three groups. Our interpretation of results is based on the confidence intervals around the point estimates, and emerging consensus [9, 12, 13] that it is mid- rather than late-life cardiometabolic factors that carry risk for dementia in late life.

A further important finding from our analyses is a higher risk of dementia in the MHO group when the threshold of 1 out of the 4 criteria is used to define unhealthy metabolic status using measures before 60 years. These results are important as the ≥ 2 out of 4 components implies that the MHO group contains individuals with both 0 and 1 adverse metabolic parameters. Redefining MHO using no adverse metabolic parameters, a stricter definition of metabolically healthy obesity supports the hypothesis that obesity before the age of 60 is associated with a higher risk of dementia, irrespective of metabolic health components (Additional file 1: Table S5). Our findings on metabolic-obesity trajectories using 3 waves of data also corroborate the main results by showing a higher risk of incident dementia in the group composed of those with persistent obesity or those who transitioned to obesity. Although the concept of MetS was not specifically elaborated for studies on dementia, a clearer picture on how best to define metabolic risk factors for dementia requires further research.

We extend findings in a previous publication from the Whitehall study using three waves spanning 10 years that also found metabolically healthy obesity to be associated with accelerated cognitive decline [29]. A study on 6030 Hispanic/Latino individuals, 50–86 years at baseline, found a greater decline over 7 years in individuals with obesity only in the presence of adverse cardiometabolic risk factors [30]. The difference in findings could be due to the nature of the study population (mostly white compared to Hispanic), the age of participants (younger at baseline in Whitehall), or the measure of obesity (BMI in Whitehall and waist circumference in the other study). It is also possible that the period over which cognitive decline is measured plays a role, 18 years in our study compared to 7 years, a period sufficiently long in our study to limit the impact of practice/learning effects.

Our findings on the association between individual cardiometabolic components with dementia are in line with previous studies showing a higher risk of dementia among persons with elevated blood pressure [6, 23, 31, 32], low HDL-C [6, 23, 31, 32], and elevated fasting glucose [6, 23, 31, 32], but our results add evidence on the importance of age at measurement of these components. The mechanisms underlying the association between metabolic-obesity phenotypes and the incidence of dementia are likely to involve those related to the components used to define the phenotypes. Obesity is thought to play a role through several pathological processes such as insulin resistance, inflammation, and mitochondrial dysfunction, contributing to neurodegeneration [33]. An adverse cardiometabolic profile is likely to exert a role through multiple processes that lead to vascular injury and neurodegeneration, driven by atherosclerotic lesions and/or microvascular dysfunction [34]. It has been suggested that brain metabolic dysfunction is the primary driver of Alzheimer’s disease [35], making impairments in insulin signaling important for neurocognitive outcomes [36]. Microvascular dysfunction due to high blood pressure is also likely to lead to oxidative stress, inflammatory responses, and increased blood–brain permeability and altered blood flow regulation [37]. Besides reverse causation in studies that measure metabolic-obesity phenotypes late in life, our findings suggest that a longer duration of exposure to cardiometabolic risk may also play a role.

The main strength of the present study is the multiple waves of data on metabolic-obesity phenotypes from midlife to late-life, allowing the examination of the role of age at assessment of metabolic-obesity phenotypes in associations with incident dementia. The use of both cognitive decline over 18 years and incident dementia also allows a more nuanced look at the validity of the concept of MHO.

The limitations of the study concern the study participants being healthier than the general population as the Whitehall II study is an occupational cohort, but risk factors-disease outcome associations in this cohort have previously shown to be similar to that in the general population [38]. The cognitive test battery did not include all possible cognitive domains, but it has been shown to be able to detect accelerated cognitive decline in those who go on to develop dementia [39]. The ascertainment of dementia using linkage to electronic health records is likely to miss milder cases of dementia and the data on dementia subtypes is incomplete, not allowing analyses on dementia subtypes. The advantage of this ascertainment method is analyses on all participants with data on metabolic-obesity phenotypes rather than only those who continue to participate in clinical examinations over the course of the study. The analysis at age ≥ 70 years is based on smaller numbers and wider CIs for the HRs compared with analysis at ages < 60 and 60 to < 70 years; nonetheless, the number of events does not violate the guideline of at least 10 events per predictor. A further limitation, as in all observational studies is residual confounding despite adjustment for several covariates in the analyses.

## Conclusions

The number of people living with dementia is projected to increase due to population aging, leading to an unsustainable increase in the burden on healthcare systems [40]. Therapeutic solutions to slow the progression of dementia remain elusive, making prevention important. Our study shows the value of careful consideration of age at assessment of putative risk factors. It also shows that while the concept of metabolically healthy obesity may be attractive due to the rapid increase in the prevalence of obesity, it is not a benign condition. Obesity in midlife is associated with accelerated cognitive decline and a higher risk of dementia at older ages, irrespective of metabolic health status.

### Supplementary Information

**Additional file 1:Table S1.** Association of individual components used to define metabolic-obesity phenotypes, measured at <60, 60 to <70, and ≥70 years, with incidence of dementia. **Table S2.** Association of metabolic-obesity phenotypes measured at <60, 60 to <70, and ≥70 years with incidence of dementia using inverse probability weighting to account for missing data. **Table S3.** Association of metabolic-obesity phenotypes measured at <60, 60 to <70, and ≥70 years with incidence of dementia excluding non-white participants from analyses. **Table S4.** Association of metabolic-obesity phenotypes measured at <60, 60 to <70, and ≥70 years with incidence of dementia excluding prevalent cases of CVD at baseline from the analyses. **Table S5.** Role of an alternative threshold used to define metabolic abnormality (≥1 unhealthy component(s)) for the association between metabolic-obesity phenotypes and incidence of dementia. **Table S6.** Transitions in the MHO phenotype group across three age strata. **Table S7.** Characteristics of participants in 1997 according to metabolic-obesity phenotypes. **Table S8.** Estimation of trajectories of metabolic-obesity phenotypes using data from 1991, 1997 and 2002: model fit statistics (group-based trajectory models). **Table S9.** Number of participants in the metabolic-obesity phenotypes as a function of trajectories using data from 1991, 1997 and 2002. **Figure S1.** Flow chart of sample selection for analyses on incident dementia. **Figure S2.** Flow chart of sample selection for analyses on cognitive decline. **Figure S3.** Trajectories of metabolic-obesity phenotypes using data from 1991, 1997 and 2002.

## Data Availability

Whitehall II data are not accessible for download because of constraints dictated by the study's ethics approval and IRB restrictions. The Whitehall II data are available for sharing within the scientific community. Researchers can apply for data access either via Dementia Platform UK https://www.dementiasplatform.uk/) or the Whitehall data sharing portal at https://www.ucl.ac.uk/epidemiology-health-care/research/epidemiology-and-public-health/research/whitehall-ii/data-sharing. For the present article, access to data was available as the last author (A Singh-Manoux) is one of the PIs of the Whitehall II study.

## Abbreviations

ADNI: Alzheimer’s Disease Neuroimaging Initiative
BMI: Body mass index
CHD: Coronary heart disease
CI: Confidence interval
CVD: Cardiovascular disease
HDL-C: High-density lipoprotein cholesterol
HES: Hospital Episode Statistics
HR: Hazard ratio
MetS: Metabolic syndrome
MHNO: Metabolically healthy non-obesity
MHO: Metabolically healthy obesity
MHSDS: Mental Health Services Data Set
MUNO: Metabolically unhealthy non-obesity
MUO: Metabolically unhealthy obesity
NHS: National Health Service
SD: Standard deviation
