# Supplementary material for: Is metabolic-healthy obesity associated with risk of dementia? An age-stratified analysis of the Whitehall II cohort study
Source: BMC Med. 2023 Nov 14;21:436. doi: 10.1186/s12916-023-03155-4 (PMC10644649; doi:10.1186/s12916-023-03155-4)

**Is metabolic-healthy obesity associated with risk of dementia? An age-stratified analysis of the Whitehall II cohort study**

Marcos D. Machado-Fragua

Séverine Sabia

Aurore Fayosse

Céline Ben Hassen

Frank van der Heide

Mika Kivimaki

Archana Singh-Manoux

**Additional file 1**

**Table S1. Association of individual components used to define metabolic-obesity phenotypes, measured at <60, 60 to <70, and ≥70 years, with incidence of dementia.**

**Table S2. Association of metabolic-obesity phenotypes** **measured** **at <60, 60 to <70, and ≥70 years with incidence of dementia using inverse probability weighting to account for missing data.**

**Table S3. Association of metabolic-obesity phenotypes** **measured** **at <60, 60 to <70, and ≥70 years with incidence of dementia excluding non-white participants from analyses.**

**Table S4. Association of metabolic-obesity phenotypes** **measured** **at <60, 60 to <70, and ≥70 years with incidence of dementia excluding prevalent cases of CVD at baseline from the analyses.**

**Table S5. Role of an alternative threshold used to define metabolic abnormality (≥1 unhealthy component(s)) for the association between metabolic-obesity phenotypes** **and incidence of dementia.**

**Table S6. Transitions in the MHO phenotype group across three age strata.**

**Table S7.** **Characteristics of participants in 1997 according to metabolic-obesity phenotypes.**

**Table S8. Estimation of trajectories of metabolic-obesity phenotypes using data from 1991, 1997 and 2002: model fit statistics (group-based trajectory models).**

**Table S9. Number of participants in the metabolic-obesity phenotypes as a function of trajectories using data from 1991, 1997 and 2002.**

**Figure S1. Flow chart of sample selection for analyses on incident dementia.**

**Figure S2. Flow chart of sample selection for analyses on cognitive decline.**

**Figure S3. Trajectories of metabolic-obesity phenotypes using data from 1991, 1997, and 2002.**

**Table S1. Association of individual components used to define metabolic-obesity phenotypes, measured at <60, 60 to <70, and ≥70 years, with incidence of dementia.**

|  | | **N Dementia cases/Total** | **HR (95% CI)** | | |
| --- | --- | --- | --- | --- | --- |
|  |  |  | **Model 1^a^** | **Model 2^b^** | **Model 3^c^** |
| **At age <60,^d^** **Median (IQR) follow-up 20.8 (15.5, 26.4) years** | | | | |  |
| Obesity non-obesity | | 340/6016 | Ref | Ref | Ref. |
| obesity | | 70/1093 | 1.46 (1.12, 1.90) | 1.41 (1.08, 1.85) | 1.31 (1.00, 1.73) |
| Metabolic Status healthy | | 250/4804 | Ref | Ref | Ref. |
| unhealthy | | 160/2305 | 1.38 (1.13, 1.69) | 1.33 (1.08, 1.62) | 1.27 (1.02, 1.57) |
| **At age 60y to <70,^e^** **Median (IQR) follow-up 10.3 (6.3, 15.4) years** | | | | | |
| Obesity non-obesity | | 310/5184 | Ref | Ref | Ref. |
| Obesity | | 69/1228 | 1.21 (0.93, 1.58) | 1.16 (0.89, 1.52) | 1.10 (0.83, 1.44) |
| Metabolic Status healthy | | 206/3550 | Ref | Ref | Ref. |
| unhealthy | | 173/2862 | 1.34 (1.09, 1.64) | 1.32 (1.07, 1.62) | 1.33 (1.07, 1.64) |
| **At age ≥70,^f^** **Median (IQR) follow-up 4.2 (3.1, 7.1) years** | | | | | |
| Obesity non-obesity | | 204/2833 | Ref | Ref | Ref. |
| obesity | | 58/731 | 1.12 (0.83, 1.52) | 1.05 (0.78, 1.43) | 1.03 (0.75, 1.40) |
| Metabolic Status healthy | | 97/1435 | Ref | Ref | Ref. |
| unhealthy | | 165/2129 | 1.19 (0.92, 1.53) | 1.17 (0.91, 1.50) | 1.20 (0.92, 1.55) |
| **Components used to measure metabolic health** | | | | | |
| **At age <60,^d^** **Median (IQR) follow-up 20.8 (15.5, 26.4) years** | | | | | |
|  | Blood pressure healthy | 193/3922 | Ref | Ref | Ref |
|  | unhealthy | 217/3187 | 1.33 (1.10, 1.62) | 1.31 (1.07, 1.59) | 1.29 (1.06, 1.58) |
|  | Triglycerides healthy | 283/4925 | Ref | Ref | Ref |
|  | unhealthy | 127/2184 | 1.07 (0.87, 1.32) | 1.01 (0.82, 1.25) | 0.86 (0.68, 1.09) |
|  | HDL-cholesterol healthy | 327/6040 | Ref | Ref | Ref |
|  | unhealthy | 83/1069 | 1.40 (1.10, 1.79) | 1.37 (1.07, 1.75) | 1.41 (1.08, 1.84) |
|  | Fasting glucose healthy | 311/5472 | Ref | Ref | Ref |
|  | unhealthy | 98/1636 | 1.14 (0.90, 1.43) | 1.13 (0.90, 1.43) | 1.09 (0.87, 1.38) |
| **At age 60y to <70,^e^** **Median (IQR) follow-up 10.3 (6.3, 15.4) years** | | | | |  |
|  | Blood pressure healthy | 157/2656 | Ref | Ref | Ref |
|  | unhealthy | 222/3756 | 0.96 (0.78, 1.18) | 0.95 (0.77, 1.17) | 0.89 (0.72, 1.10) |
|  | Triglycerides healthy | 241/3853 | Ref | Ref | Ref |
|  | unhealthy | 138/2559 | 1.20 (0.97, 1.49) | 1.16 (0.94, 1.44) | 0.98 (0.76, 1.28) |
|  | HDL-cholesterol healthy | 277/4507 | Ref | Ref | Ref |
|  | unhealthy | 102/1904 | 1.41 (1.12, 1.79) | 1.35 (1.07, 1.71) | 1.33 (1.00, 1.76) |
|  | Fasting glucose healthy | 265/4730 | Ref | Ref | Ref |
|  | unhealthy | 114/1682 | 1.48 (1.18, 1.86) | 1.48 (1.18, 1.86) | 1.46 (1.16, 1.85) |
| **At age ≥70,^f^** **Median (IQR) follow-up 4.2 (3.1, 7.1) years** | | | | |  |
|  | Blood pressure healthy | 67/983 | Ref | Ref | Ref |
|  | unhealthy | 195/2581 | 1.01 (0.76, 1.33) | 0.97 (0.73, 1.28) | 0.90 (0.68, 1.21) |
|  | Triglycerides healthy | 119/1650 | Ref | Ref | Ref |
|  | unhealthy | 143/1914 | 1.09 (0.86, 1.40) | 1.06 (0.83, 1.36) | 0.93 (0.57, 1.51) |
|  | HDL-cholesterol healthy | 133/1816 | Ref | Ref | Ref |
|  | unhealthy | 129/1748 | 1.10 (0.86, 1.41) | 1.09 (0.85, 1.40) | 1.12 (0.69, 1.82) |
|  | Fasting glucose healthy | 170/2588 | Ref | Ref | Ref |
| unhealthy | unhealthy | 92/976 | 1.39 (1.07, 1.80) | 1.37 (1.06, 1.78) | 1.38 (1.06, 1.81) |

IQR: interquartile range; HR: hazard ratio; CI confidence interval

^a^Model 1: analyses adjusted for age (timescale), sex, education, ethnicity, marital status and birth cohort (5-year groups)

^b^Model 2: Model 1 plus adjustment for health-related behaviors (smoking, alcohol consumption, consumption of fruits and vegetables, and physical activity)

^c^Model 3: Model 2 plus adjustment for the other metabolic-obesity components

^d^Mean (SD) age at assessment=55.1 (2.9) years

^e^Mean (SD) age at assessment=65.0 (1.5) years

^f^Mean (SD) age at assessment=73.9 (1.9) years

**Table S2. Association of metabolic-obesity phenotypes measured at <60, 60 to <70, and ≥70 years with incidence of dementia using inverse probability weighting to account for missing data.**

|  | | | **Metabolic-obesity phenotypes** | | | |
| --- | --- | --- | --- | --- | --- | --- |
|  |  |  | **MHNO** | **MUNO** | **MHO** | **MUO** |
| **At age <60y^a^,** **Median (IQR) follow-up 20.8 (15.5, 26.4) years** | | | | | | |
|  | N Dementia cases/Total | | 217/4301 | 123/1715 | 33/503 | 37/590 |
|  | Rate/1000 person-years | | 2.44 | 3.49 | 3.80 | 3.29 |
|  | Models, HR (95% CI) | |  |  |  |  |
|  |  | Model 1^d^ | Ref. | 1.43 (1.14, 1.79) | 1.82 (1.24, 2.67) | 1.52 (1.07, 2.16) |
|  |  | Model 2^e^ | Ref. | 1.38 (1.10, 1.73) | 1.80 (1.23, 2.65) | 1.44 (1.01, 2.06) |
| **At age 60y to <70y^b^,** **Median (IQR) follow-up 10.3 (6.3, 15.4) years** | | | | | | |
|  | N Dementia cases/Total | | 183/3117 | 127/2067 | 23/433 | 46/795 |
|  | Rate/1000 person-years | | 5.41 | 6.23 | 4.95 | 7.18 |
|  | Models, HR (95% CI) | |  |  |  |  |
|  |  | Model 1^d^ | Ref. | 1.31 (1.03, 1.65) | 0.94 (0.58, 1.51) | 1.67 (1.19, 2.35) |
|  |  | Model 2^e^ | Ref. | 1.29 (1.02, 1.62) | 0.90 (0.56, 1.46) | 1.59 (1.14, 2.23) |
| **At age ≥70y^c^,** **Median (IQR) follow-up 4.2 (3.1, 7.1) years** | | | | | | |
|  | N Dementia cases/Total | | 83/1244 | 121/1589 | 14/191 | 44/540 |
|  | Rate/1000 person-years | | 13.81 | 15.70 | 15.69 | 15.74 |
|  | Models, HR (95% CI) | |  |  |  |  |
|  |  | Model 1^d^ | Ref. | 1.13 (0.84, 1.52) | 1.08 (0.58, 1.98) | 1.13 (0.76, 1.67) |
|  |  | Model 2^e^ | Ref. | 1.10 (0.82, 1.49) | 0.98 (0.54, 1.81) | 1.05 (0.71, 1.56) |

MHNO: Metabolically healthy non-obesity; MUNO: metabolically unhealthy non-obesity; MHO: metabolically healthy obesity; MUO: metabolically unhealthy obesity;

IQR: interquartile range; HR: hazard ratio: CI confidence interval

^a^ Mean (SD) age at assessment=55.1 (2.9) years

^b^ Mean (SD) age at assessment=65.0 (1.5) years

^c^ Mean (SD) age at assessment=73.9 (1.9) years

^d^ Model 1: analyses adjusted for age (timescale), sex, education, ethnicity, marital status and birth cohort (5-year groups)

^e^ Model 2: Model 1 plus adjustment for health-related behaviors (smoking, alcohol consumption, consumption of fruits and vegetables, and physical activity)

**Table S3. Association of metabolic-obesity phenotypes measured at <60, 60 to <70, and ≥70 years with incidence of dementia excluding non-white participants from analyses.**

|  | | | **Metabolic-obesity phenotypes** | | | |
| --- | --- | --- | --- | --- | --- | --- |
|  |  |  | **MHNO** | **MUNO** | **MHO** | **MUO** |
| **At age <60y^a^,** **Median (IQR) follow-up 20.7 (15.4, 26.3) years** | | | | | | |
|  | N Dementia cases/Total | | 191/3937 | 96/1504 | 28/435 | 31/522 |
|  | Rate/1000 person-years | | 2.43 | 3.27 | 3.57 | 3.31 |
|  | Models, HR (95% CI) | |  |  |  |  |
|  |  | Model 1^d^ | Ref. | 1.37 (1.06, 1.75) | 1.80 (1.20, 2.69) | 1.60 (1.10, 2.35) |
|  |  | Model 2^e^ | Ref. | 1.31 (1.02, 1.68) | 1.72 (1.15, 2.59) | 1.51 (1.03, 2.22) |
| **At age 60y to <70y^b^,** **Median (IQR) follow-up 10.2 (6.3, 15.3) years** | | | | | | |
|  | N Dementia cases/Total | | 165/2922 | 98/1835 | 21/393 | 40/719 |
|  | Rate/1000 person-years | | 5.04 | 5.25 | 5.12 | 5.99 |
|  | Models, HR (95% CI) | |  |  |  |  |
|  |  | Model 1^d^ | Ref. | 1.22 (0.95, 1.57) | 1.16 (0.73, 1.84) | 1.67 (1.17, 2.37) |
|  |  | Model 2^e^ | Ref. | 1.20 (0.93, 1.55) | 1.11 (0.70, 1.77) | 1.58 (1.11, 2.25) |
| **At age ≥70y^c^,** **Median (IQR) follow-up 4.1 (3.1, 7.1) years** | | | | | | |
|  | N Dementia cases/Total | | 70/1155 | 99/1407 | 14/175 | 40/481 |
|  | Rate/1000 person-years | | 10.34 | 12.42 | 14.62 | 14.96 |
|  | Models, HR (95% CI) | |  |  |  |  |
|  |  | Model 1^d^ | Ref. | 1.23 (0.91, 1.68) | 1.44 (0.80, 2.59) | 1.49 (1.00, 2.21) |
|  |  | Model 2^e^ | Ref. | 1.21 (0.89, 1.65) | 1.31 (0.73, 2.36) | 1.40 (0.94, 2.08) |

MHNO: Metabolically healthy non-obesity; MUNO: metabolically unhealthy non-obesity; MHO: metabolically healthy obesity; MUO: metabolically unhealthy obesity;

IQR: interquartile range; HR: hazard ratio; CI: confidence interval

^a^ Mean (SD) age at assessment=55.1 (2.9) years

^b^ Mean (SD) age at assessment=65.0 (1.5) years

^c^ Mean (SD) age at assessment=73.9 (1.9) years

^d^ Model 1: analyses adjusted for age (timescale), sex, education, ethnicity, marital status and birth cohort (5-year groups)

^e^ Model 2: Model 1 plus adjustment for health-related behaviors (smoking, alcohol consumption, consumption of fruits and vegetables, and physical activity)

**Table S4. Association of metabolic-obesity phenotypes measured at <60, 60 to <70, and ≥70 years with incidence of dementia excluding prevalent cases of CVD at baseline from the analyses.**

|  | | | | | **Metabolic-obesity phenotypes** | | | |
| --- | --- | --- | --- | --- | --- | --- | --- | --- |
|  |  |  |  |  | **MHNO** | **MUNO** | **MHO** | **MUO** |
| **At age <60y^a^,** **Median (IQR) follow-up 20.8 (15.5, 26.5) years** | | | | | | | | |
|  | N Dementia cases/Total | | | | 207/4173 | 112/1560 | 31/477 | 34/516 |
|  | Rate/1000 person-years | | | | 2.46 | 3.61 | 3.57 | 3.59 |
|  | Models, HR (95% CI) | | | |  |  |  |  |
|  |  | Model 1^d^ | | | Ref. | 1.41 (1.12, 1.79) | 1.73 (1.18, 2.54) | 1.64 (1.14, 2.36) |
|  |  | Model 2^e^ | | | Ref. | 1.36 (1.08, 1.73) | 1.70 (1.15, 2.50) | 1.56 (1.08, 2.26) |
| **At age 60y to <70y^b^,** **Median (IQR) follow-up 10.3 (6.3, 15.4) years** | | | | | | | | |
|  | N Dementia cases/Total | | | | 170/2935 | 98/1680 | 20/395 | 38/628 |
|  | Rate/1000 person-years | | | | 5.14 | 5.61 | 4.77 | 6.44 |
|  | Models, HR (95% CI) | | | |  |  |  |  |
|  |  | | Model 1^d^ | | Ref. | 1.23 (0.96, 1.59) | 1.03 (0.64, 1.65) | 1.66 (1.16, 2.37) |
|  |  | | Model 2^e^ | | Ref. | 1.24 (0.96, 1.59) | 1.01 (0.63, 1.61) | 1.61 (1.12, 2.30) |
| **At age ≥70y^c^,** **Median (IQR) follow-up 4.1 (3.2, 7.1) years** | | | | | | | | |
|  | N Dementia cases/Total | | | | 76/1125 | 76/1120 | 8/148 | 28/372 |
|  | Rate/1000 person-years | | | | 11.59 | 12.02 | 9.49 | 13.70 |
|  | Models, HR (95% CI) | | | |  |  |  |  |
|  |  | | | Model 1^d^ | Ref. | 1.05 (0.76, 1.45) | 0.76 (0.36, 1.60) | 1.15 (0.74, 1.79) |
|  |  | | | Model 2^e^ | Ref. | 1.02 (0.74, 1.40) | 0.67 (0.32, 1.40) | 1.04 (0.66, 1.62) |

CVD: cardiovascular disease (heart failure, coronary heart disease, and stroke); MHNO: Metabolically healthy non-obesity; MUNO: metabolically unhealthy non-obesity; MHO: metabolically healthy obesity; MUO: metabolically unhealthy obesity; IQR: interquartile range; HR: hazard ratio; CI: confidence interval

^a^ Mean (SD) age at assessment=55.1 (2.9) years

^b^ Mean (SD) age at assessment=65.0 (1.5) years

^c^ Mean (SD) age at assessment=73.9 (1.9) years

^d^ Model 1: analyses adjusted for age (timescale), sex, education, marital status and birth cohort (5-year groups)

^e^ Model 2: Model 1 plus adjustment for health-related behaviors (smoking, alcohol consumption, consumption of fruits and vegetables, and physical activity).

**Table S5. Role of an alternative threshold used to define metabolic abnormality (≥1 unhealthy component(s)) for the association between metabolic-obesity phenotypes and incidence of dementia.**

|  | | | **Metabolic-obesity phenotypes** | | | |
| --- | --- | --- | --- | --- | --- | --- |
|  |  |  | **MHNO** | **MUNO** | **MHO** | **MUO** |
| **At age <60y^a^,** **Median (IQR) follow-up 20.8 (15.5, 26.5) years** | | | | | | |
|  | N Dementia cases/Total | | 102/2245 | 238/3771 | 12/145 | 58/948 |
|  | Rate/1000 person-years | | 2.26 | 3.16 | 4.53 | 3.38 |
|  | Models, HR (95% CI) | |  |  |  |  |
|  |  | Model 1^d^ | Ref. | 1.34 (1.06, 1.70) | 2.09 (1.14, 3.82) | 1.71 (1.23, 2.36) |
|  |  | Model 2^e^ | Ref. | 1.28 (1.01, 1.62) | 2.04 (1.11, 3.74) | 1.60 (1.15, 2.22) |
| **At age 60y to <70y^b^,** **Median (IQR) follow-up 10.3 (6.3, 15.4) years** | | | | | | |
|  | N Dementia cases/Total | | 74/1443 | 236/3741 | 5/115 | 64/1113 |
|  | Rate/1000 person-years | | 4.63 | 5.80 | 4.48 | 5.83 |
|  | Models, HR (95% CI) | |  |  |  |  |
|  |  | Model 1^d^ | Ref. | 1.29 (0.99, 1.68) | NA | 1.51 (1.07, 2.12) |
|  |  | Model 2^e^ | Ref. | 1.28 (0.99, 1.67) | NA | 1.43 (1.01, 2.01) |
| **At age ≥70y^c^,** **Median (IQR) follow-up 4.1 (3.2, 7.1) years** | | | | | | |
|  | N Dementia cases/Total | | 26/490 | 178/2343 | 5/41 | 53/690 |
|  | Rate/1000 person-years | | 9.22 | 13.11 | 23.33 | 13.75 |
|  | Models, HR (95% CI) | |  |  |  |  |
|  |  | Model 1^d^ | Ref. | 1.39 (0.92, 2.11) | NA | 1.42 (0.88, 2.29) |
|  |  | Model 2^e^ | Ref. | 1.34 (0.88, 2.02) | NA | 1.29 (0.80, 2.09) |

MHNO: Metabolically healthy non-obesity; MUNO: metabolically unhealthy non-obesity; MHO: metabolically healthy obesity; MUO: metabolically unhealthy obesity; IQR: interquartile range; HR: hazard ratio; CI: confidence interval

NA: not applicable (insufficient number of cases (≤5) to allow analysis)

^a^ Mean (SD) age at assessment=55.1 (2.9) years

^b^ Mean (SD) age at assessment=65.0 (1.5) years

^c^ Mean (SD) age at assessment=73.9 (1.9) years

^d^ Model 1: analyses adjusted for age (timescale), sex, education, marital status and birth cohort (5-year groups)

^e^ Model 2: Model 1 plus adjustment for health-related behaviors (smoking, alcohol consumption, consumption of fruits and vegetables, and physical activity)

**Table S6. Transitions in the MHO phenotype group across three age strata.**

|  | **MHO phenotype at <60 years** | **MHO phenotype at 60 to <70 years** |
| --- | --- | --- |
|  | **Outcome at 60 to <70 years**  **(N=503)** | **Outcome at ≥70 years**  **(N=433)** |
|  | **N (%)** | **N (%)** |
| **Remain MHO** | 154 (30.6) | 88 (20.3) |
| **Transition to MHNO** | 48 (9.5) | 17 (3.9) |
| **Transition to MUNO** | 24 (4.8) | 17 (3.9) |
| **Transition to MUO** | 158 (31.4) | 102 (23.6) |
| **Death** | 44 (8.7) | 30 (6.9) |
| **Dementia** | 13 (2.6) | 5 (1.2) |
| **No data on metabolic-obesity phenotype** | 62 (12.3) | 174 (40.2) |

MHNO: Metabolically healthy non-obesity; MUNO: metabolically unhealthy non-obesity; MHO: metabolically healthy obesity;

MUO: metabolically unhealthy obesity

**Table S7. Characteristics of participants in 1997 according to metabolic-obesity phenotypes.**

|  | | **Metabolic-obesity phenotypes** | | | | ***p*-value** |
| --- | --- | --- | --- | --- | --- | --- |
|  |  | **MHNO** | **MUNO** | **MHO** | **MUO** |  |
| Age, years, M (SD) | | 55.3 (6.0) | 57.1 (6.0) | 55.5 (5.8) | 56.1 (5.8) | <0.01 |
| Sex, women | | 1446 (30.6) | 267 (18.2) | 232 (48.2) | 178 (36.8) | <0.01 |
| Education, low | | 2009 (42.5) | 692 (47.2) | 234 (48.6) | 231 (47.7) | <0.01 |
| Ethnicity, non-white | | 343 (7.3) | 158 (10.8) | 56 (11.6) | 49 (10.1) | <0.01 |
| Marital status, married | | 3657 (77.4) | 1175 (80.2) | 336 (69.9) | 357 (73.8) | <0.01 |
| Smoking, current smokers | | 482 (10.2) | 156 (10.6) | 45 (9.4) | 44 (9.1) | <0.01 |
| Alcohol, moderate drinkers | | 2493 (52.8) | 688 (47.0) | 214 (44.5) | 209 (43.2) | <0.01 |
| Fruits and vegetables, ≥2/day | | 1743 (36.9) | 504 (34.4) | 200 (41.6) | 169 (34.9) | 0.02 |
| Moderate-vigorous physical activity, h/week, M(SD) | | 3.4 (3.2) | 3.5 (3.4) | 2.8 (2.8) | 2.5 (2.7) | <0.01 |
| Prevalence of CVD | | 189 (4.0) | 179 (12.2) | 30 (6.2) | 67 (13.8) | <0.01 |
| Body mass index, kg/m², M(SD) | | 24.6 (2.6) | 26.1 (2.2) | 33.0 (2.9) | 33.5 (3.4) | <0.01 |
| **Metabolic components** | |  |  |  |  |  |
|  | Elevated triglycerides | 390 (8.6) | 915 (64.2) | 57 (12.1) | 316 (68.0) | <0.01 |
|  | Low HDL-C | 164 (3.6) | 461 (32.4) | 20 (4.2) | 162 (34.8) | <0.01 |
|  | Elevated blood pressure | 1226 (26.8) | 1028 (72.0) | 231 (48.9) | 367 (78.8) | <0.01 |
|  | Elevated fasting glucose | 300 (6.6) | 549 (38.5) | 25 (5.3) | 202 (43.4) | <0.01 |
| **Standardized cognitive test scores, M(SD)** | | |  |  |  |  |
|  | Global cognitive score | 0.06 (1.0) | -0.16 (1.0) | -0.04 (1.0) | -0.14 (1.1) | <0.01 |
|  | Memory | 0.06 (1.0) | -0.16 (0.9) | 0.02 (1.0) | -0.12 (0.9) | <0.01 |
|  | Reasoning | 0.05 (0.9) | -0.06 (1.1) | -0.10 (1.1) | -0.16 (1.1) | <0.01 |
|  | Phonemic fluency | 0.04 (1.0) | -0.11 (1.0) | -0.07 (1.0) | -0.05 (1.0) | <0.01 |
|  | Semantic fluency | 0.05 (1.0) | -0.14 (1.0) | 0.01 (1.1) | -0.12 (1.1) | <0.01 |

MHNO: metabolically healthy non-obesity; MUNO: metabolically unhealthy non-obesity; MHO: metabolically healthy obesity; MUO: metabolically unhealthy obesity; M: mean; SD: standard deviation; CVD: cardiovascular disease (stroke, coronary heart disease, and heart failure); HDL-C: high-density lipoprotein cholesterol. Data are n (%), unless otherwise specified

**Table S8. Estimation of trajectories of metabolic-obesity phenotypes using data from 1991, 1997 and 2002: model fit statistics (group-based trajectory models).^a^**

| **Number of groups**^b^ | **Trajectory**  **Shape^c^** | **Allocated**  **Group Membership** | **BIC^d^** | **Average Posterior**  **Probabilities**^e^ | **AIC**^f^ |
| --- | --- | --- | --- | --- | --- |
| 4 | 1  1  1  1 | 42.0  12.3  36.3  9.4 | -18318.13 | 0.86  0.72  0.89  0.90 | -18277.19 |
| 4 | 1  2  2  2 | 37.6  18.9  34.1  9.4 | -18349.39 | 0.84  0.67  0.85  0.90 | -18298.22 |
| 4 | 2  2  2  2 | 37.6  18.9  34.1  9.4 | -18353.48 | 0.84  0.67  0.85  0.90 | -18299.22 |
| 4 | 2  2  2  1 | 42.5  12.1  9.4  36.0 | -18323.97 | 0.87  0.73  0.91  0.89 | -18272.80 |
| 5 | 1  1  2  2  2 | 6.0  12.2  38.1  34.2  9.5 | -18250.42 | 0.73  0.76  0.85  0.90  0.92 | -18189.02 |
| **5** | **2**  **2**  **2**  **2**  **2** | **38.6**  **6.0**  **12.0**  **33.9**  **9.5** | **-18249.36** | **0.86**  **0.73**  **0.78**  **0.89**  **0.92** | **-18181.13** |

^a^ Only models identifying groups with an allocated group membership ≥5% of the total sample are shown.

^b^ Number of trajectory groups estimated. Although we tested the models with 6 trajectories, none of the models fits the criteria presented in ^a^, so data are not displayed for models with 6 trajectories.

**^c^** Polynomial function of time (0 intercept only, 1 linear, 2 quadratic).

**^d^** Bayesian Information Criterion (BIC), a difference of 10 is strong evidence that the model with the lowest BIC (compared to null) has best fit.

^e^ Posterior probabilities of group membership for individuals assigned to each group, an average > 0.7 demonstrates good classification accuracy.

^f^ Akaike Information Criterion (AIC).

Model selected based on fulfilment of criteria ^§^ and evidence of improved fit using lowest BIC/AIC score.

**Table S9. Number of participants in the metabolic-obesity phenotypes as a function of trajectories using data from 1991, 1997 and 2002.**

|  | | **Trajectory 1**  **Persistent MHNO** | **Trajectory 2**  **Transition from MUNO to MHNO** | **Trajectory 3**  **Transition from MHNO to MUNO** | **Trajectory 4**  **Persistent/transition to unhealthy metabolic status and/or transition to obesity** | **Trajectory 5**  **Transition to obesity and persistent obesity** |
| --- | --- | --- | --- | --- | --- | --- |
| **N** | | 3048 | 321 | 603 | 2195 | 622 |
| **1991** | |  |  |  |  |  |
|  | MHNO | 2947 (100.0) | 0 (0.0) | 559 (100.0) | 858 (40.3) | 28 (4.7) |
|  | MUNO | 0 (0.0) | 307 (95.6) | 0 (0.0) | 1110 (52.1) | 150 (25.1) |
|  | MHO | 0 (0.0) | 8 (2.5) | 0 (0.0) | 136 (6.4) | 111 (18.5) |
|  | MUO | 0 (0.0) | 6 (1.9) | 0 (0.0) | 26 (1.2) | 309 (51.7) |
| **1997** | |  |  |  |  |  |
|  | MHNO | 2477 (100.0) | 227 (100.0) | 603 (100.0) | 308 (18.0) | 4 (0.9) |
|  | MUNO | 0 (0.0) | 0 (0.0) | 0 (0.0) | 1069 (62.5) | 9 (1.9) |
|  | MHO | 0 (0.0) | 0 (0.0) | 0 (0.0) | 288 (16.8) | 124 (26.5) |
|  | MUO | 0 (0.0) | 0 (0.0) | 0 (0.0) | 46 (2.7) | 331 (70.7) |
| **2002** | |  |  |  |  |  |
|  | MHNO | 2732 (100.0) | 321 (100.0) | 0 (0.0) | 262 (13.3) | 0 (0.0) |
|  | MUNO | 0 (0.0) | 0 (0.0) | 481 (79.8) | 1230 (62.2) | 7 (1.2) |
|  | MHO | 0 (0.0) | 0 (0.0) | 84 (13.9) | 296 (15.0) | 86 (15.1) |
|  | MUO | 0 (0.0) | 0 (0.0) | 38 (6.3) | 188 (9.5) | 476 (83.7) |

MHNO: metabolically healthy non-obesity; MUNO: metabolically unhealthy non-obesity; MHO: metabolically healthy obesity; MUO: metabolically unhealthy obesity;

**Figure S1. Flow chart of sample selection for analyses on incident dementia.**


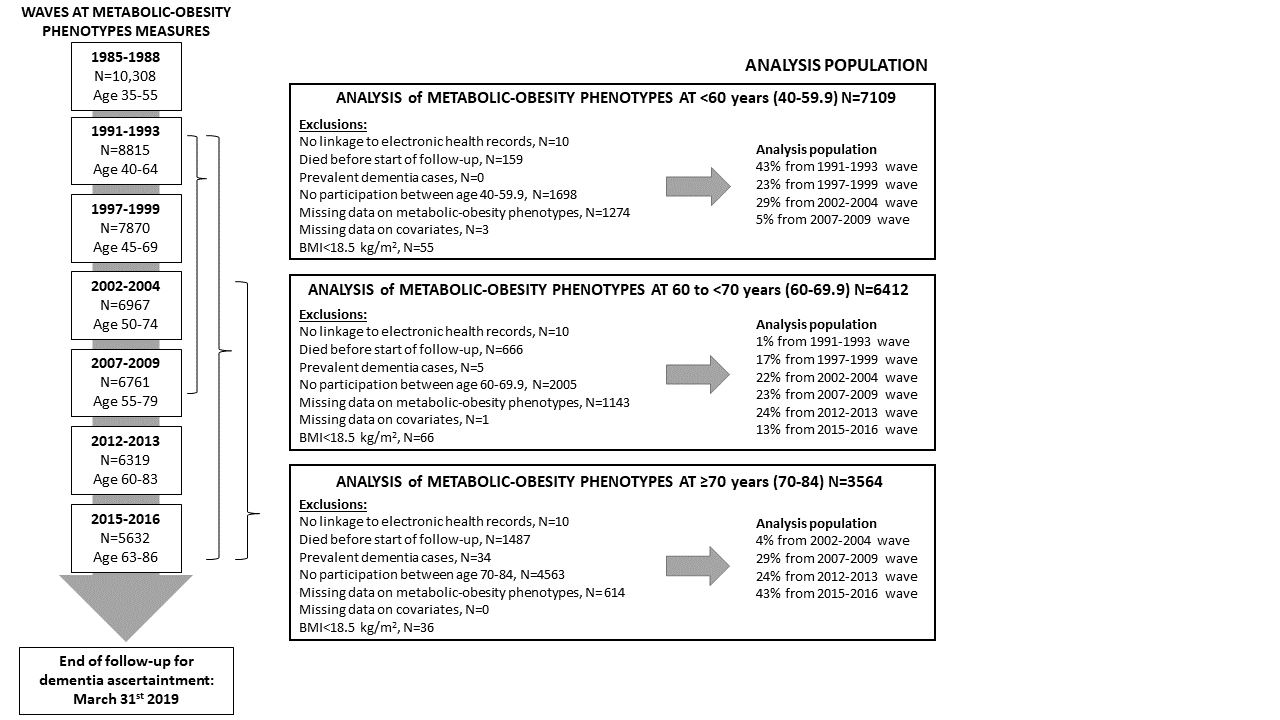


**Figure S2. Flow chart of sample selection for analyses on cognitive decline.**

**Whitehall II Study, 1985-1988**

N=10308

**Excluded, 1985 to 1997**

- Incident dementia, N=1

- Deaths, N=306

- Lost to follow-up, N= 1904

**Participants at baseline, 1997-1999**

N=8097

**Missing data**

- Metabolic-obesity phenotypes, N=406

- Cognitive tests, N=531

- Covariates, N=4

**Main cognitive function analyses**

Participants in the analyses, N=7156 *

Follow-up waves for cognitive decline,

2002-2004, 2007-2009, 2012-2013 and 2015-2016

*3480 (48.6%) had cognitive data at all 5 waves (1997, 2002, 2007, 2012, 2015), 1351 (18.9%) at 4 waves, 832 (11.6%) at 3 waves, 716 (10.0%) at 2 waves, and 777 (10.9%) at one wave.

**Figure S3. Trajectories of metabolic-obesity phenotypes using data from 1991, 1997, and 2002.**


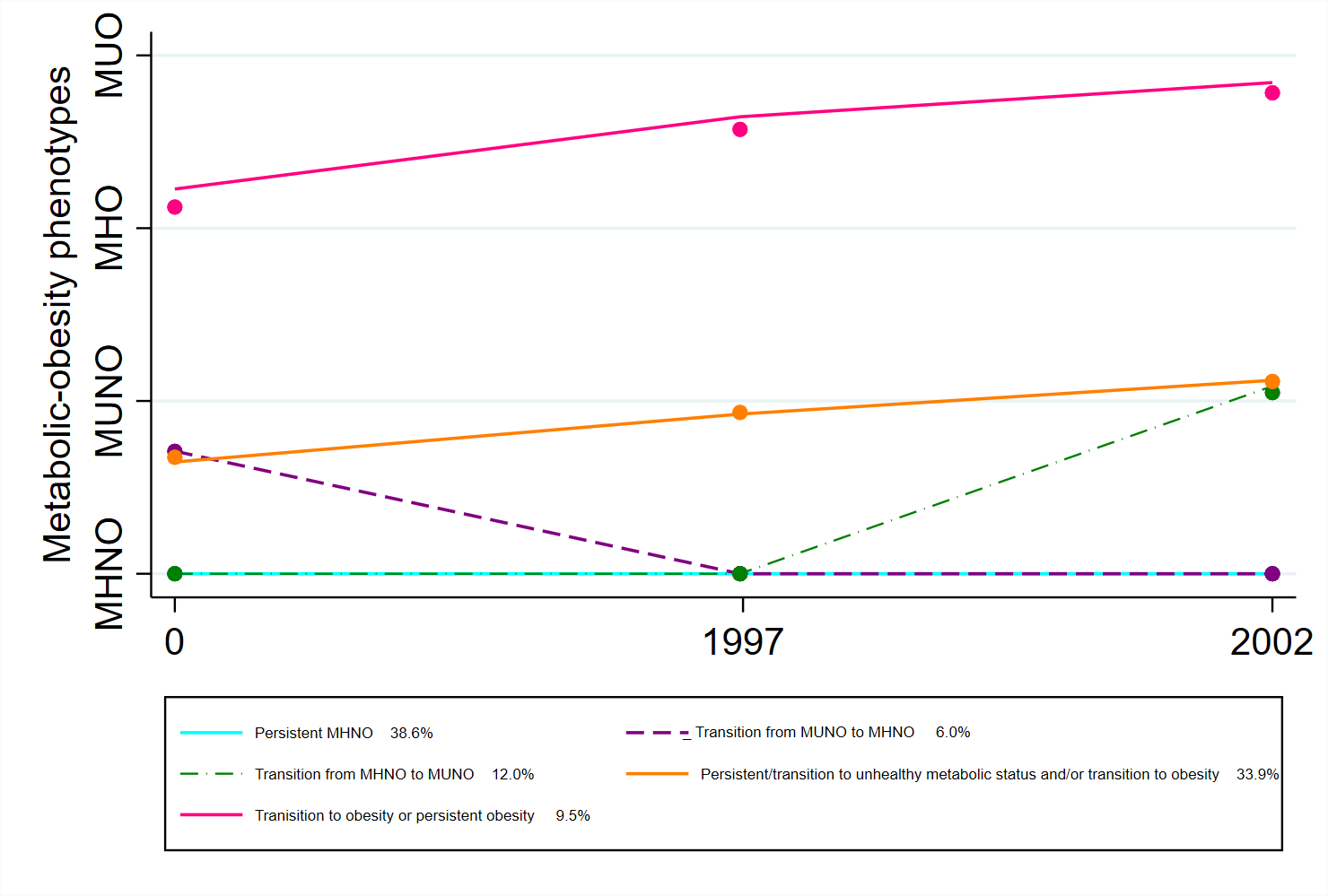

Supplement: Supplementary file 1 — Additional file 1:Table S1. Association of individual components used to define metabolic-obesity phenotypes, measured at <60, 60 to <70, and ≥70 years, with incidence of dementia. Table S2. Association of metabolic-obesity phenotypes measured at <60, 60 to <70, and ≥70 years with incidence of dementia using inverse probability weighting to account for missing data. Table S3. Association of metabolic-obesity phenotypes measured at <60, 60 to <70, and ≥70 years with incidence of dementia excluding non-white participants from analyses. Table S4. Association of metabolic-obesity phenotypes measured at <60, 60 to <70, and ≥70 years with incidence of dementia excluding prevalent cases of CVD at baseline from the analyses. Table S5. Role of an alternative threshold used to define metabolic abnormality (≥1 unhealthy component(s)) for the association between metabolic-obesity phenotypes and incidence of dementia. Table S6. Transitions in the MHO phenotype group across three age strata. Table S7. Characteristics of participants in 1997 according to metabolic-obesity phenotypes. Table S8. Estimation of trajectories of metabolic-obesity phenotypes using data from 1991, 1997 and 2002: model fit statistics (group-based trajectory models). Table S9. Number of participants in the metabolic-obesity phenotypes as a function of trajectories using data from 1991, 1997 and 2002. Figure S1. Flow chart of sample selection for analyses on incident dementia. Figure S2. Flow chart of sample selection for analyses on cognitive decline. Figure S3. Trajectories of metabolic-obesity phenotypes using data from 1991, 1997 and 2002. [file 12916_2023_3155_MOESM1_ESM.docx]
